# Endothelial glycocalyx degradation is more severe in patients with non-pulmonary sepsis compared to pulmonary sepsis and associates with risk of ARDS and other organ dysfunction

**DOI:** 10.1186/s13613-017-0325-y

**Published:** 2017-10-06

**Authors:** Laura S. Murphy, Nancy Wickersham, J. Brennan McNeil, Ciara M. Shaver, Addison K. May, Julie A. Bastarache, Lorraine B. Ware

**Affiliations:** 1 Vanderbilt University School of Medicine, Nashville, TN USA; 2 Division of Allergy, Pulmonary and Critical Care Medicine, Department of Medicine, Vanderbilt University Medical Center, Vanderbilt University School of Medicine, T1218 Medical Center North, 1161 21st Avenue S, Nashville, TN 37232-2650 USA; 3 Department of Surgery, Vanderbilt University Medical Center, Nashville, TN USA; 4 Department of Pathology, Microbiology and Immunology, Vanderbilt University School of Medicine, Nashville, TN USA

**Keywords:** Syndecan-1, Glycocalyx, Sepsis, Acute respiratory distress syndrome (ARDS)

## Abstract

**Background:**

Disruption of the endothelial glycocalyx contributes to acute lung injury in experimental sepsis but has not been well studied in humans. To study glycocalyx degradation in sepsis-induced ARDS, we measured plasma levels of syndecan-1, a marker for glycocalyx degradation.

**Methods:**

The present study is a retrospective observational study of 262 ventilated medical ICU patients at risk of ARDS due to severe sepsis and APACHE II ≥ 25. Plasma syndecan-1 was measured at study enrollment. Primary analysis focused on the association between syndecan-1 levels and the development of ARDS, other organ dysfunction (Brussels criteria), or in-hospital mortality.

**Results:**

Overall, 135 (52%) patients developed ARDS. In patients with non-pulmonary sepsis, syndecan-1 levels were associated with ARDS (*p* = 0.05). Regardless of etiology of sepsis, higher syndecan-1 levels were associated with hepatic (*p* < 0.001), renal (*p* = 0.003), coagulation (*p* = 0.001), and circulatory (*p* = 0.02) failure as well as in-hospital mortality (*p* = 0.001), and there was a significant association between syndecan-1 levels and the number of vasopressors required in the first 24 h (*p* < 0.001). In addition, elevated syndecan levels were independently predictive of mortality in multivariable logistic regression adjusted for age and APACHE II score (odds ratio 1.85 per log increase in syndecan-1, 95% CI 1.056–3.241, *p* = 0.03).

**Conclusion:**

The extent of endothelial glycocalyx degradation is associated with non-pulmonary organ dysfunction in subjects with sepsis and is associated with ARDS but only in the subgroup with non-pulmonary sepsis. Measurement of syndecan-1 levels in sepsis patients might be useful for identifying patients at high risk of organ dysfunction and mortality as well as those who could benefit from therapies targeted at protecting or restoring the glycocalyx.

## Background

The endothelial glycocalyx is a complex layer of glycoproteins, proteoglycans, and glycosaminoglycans that coats the luminal surface of the microvascular endothelium. Hydrated glycosaminoglycans form a thick and rigid endothelial surface layer (ESL) that plays a key role in limiting vascular permeability and regulating leukocyte adhesion [1, 2]. Shedding of the ESL occurs in response to a variety of insults and results in hyperpermeability, inappropriate leukocyte adhesion [3], and loss of capillary autoregulation [4].

The major endothelial cell surface proteoglycans are syndecans, which are heparan sulfate proteoglycans. Syndecan-1 is abundant in the endothelial surface layer, and circulating syndecan-1 is a marker of endothelial glycocalyx degradation [5–11]. In mice, plasma syndecan-1 levels are negatively correlated with ESL thickness and positively correlated with microvascular permeability [12]. Glycocalyx dysfunction and syndecan-1 shedding have been described in a variety of clinical pathophysiologic processes, including sepsis [13], hemorrhagic shock [14], atherosclerosis [15], acute coronary syndrome [16], renal disease [17], diabetes [10], and hypervolemia [18]. Furthermore, elevated plasma syndecan-1 levels have been associated with increased mortality in patients with trauma and sepsis [8, 11]. However, despite the known role of endothelial injury and activation in the pathogenesis of ARDS [19–23], the association between syndecan-1 and development of ARDS or other organ dysfunction in sepsis has not been well studied.

Both pulmonary and non-pulmonary sepsis can lead to ARDS with pulmonary sepsis (due to pneumonia or aspiration of gastric contents) resulting in direct injury to the lung and non-pulmonary sepsis resulting in indirect injury to the lung [22]. In both animal models and patients, direct injury to the lung is characterized by more severe lung epithelial damage, and an indirect insult to the lung is characterized by more severe systemic endothelial injury [24–26]. However, whether there is greater disruption of the glycocalyx in indirect versus direct ARDS has not been well studied. In a mouse model of non-pulmonary sepsis, glycocalyx degradation contributed to acute lung injury, and human studies have shown an association between glycocalyx degradation and development of pulmonary edema [27, 28], findings that support an association between glycocalyx degradation and ARDS. In addition, a study of 17 patients with acute respiratory failure demonstrated differences in the overall pattern of circulating glycosaminoglycans between those with direct versus indirect causes of acute respiratory failure, raising the question of whether glycocalyx degradation differs depending on the mechanism of lung injury. However, syndecan-1 was not measured in that study and it was not reported whether the patients met clinical criteria for ARDS [29].

To address these gaps in knowledge, we designed the current study to test the hypothesis that in critically ill patients with sepsis, the extent of glycocalyx disruption as measured by plasma syndecan-1 levels is associated with development of ARDS and that glycocalyx degradation is more extensive in non-pulmonary sepsis compared to pulmonary sepsis. We further hypothesized that the degree of glycocalyx degradation is associated with other organ dysfunction and adverse clinical outcomes including in-hospital mortality. Finally, we hypothesized that neutrophil activation may contribute to enzymatic cleavage of syndecan-1 from the surface of endothelial cells, and we tested this hypothesis by measuring myeloperoxidase (MPO), a circulating marker of neutrophil activation [26].

## Methods

### Study design and patient selection

We conducted a retrospective observational study within the validating acute lung injury biomarkers for diagnosis (VALID) study. VALID is a prospective cohort study enrolling critically ill patients in the Vanderbilt Medical, Surgical, Trauma and Cardiovascular ICUs since 2006 [30]. Patients are enrolled on the morning of ICU day 2 if they are not being transferred out of ICU on that day. At the time of enrollment, plasma is obtained for biomarker measurement. Comprehensive clinical data are collected for the first four ICU days including acute physiology and chronic health evaluation II (APACHE II) [31], daily laboratory values, hemodynamics, ventilator settings, medications, and daily phenotyping for severe sepsis, ARDS, and other organ failures. The VALID study is approved by the Vanderbilt Institutional Review Board. Informed consent is obtained from patients or their surrogates if possible; the Institutional Review Board has also granted a waiver of informed consent for this minimal risk study.

For the current study, medical ICU patients enrolled in the VALID study who met Sepsis-2 criteria for severe sepsis [32] were included if they were at high risk of ARDS based on APACHE II score ≥ 25 and need for mechanical ventilation and had citrated plasma available from enrollment day for measurement of syndecan-1. Patients with pneumonia or aspiration of gastric contents were classified as pulmonary sepsis, and the remaining patients were classified as non-pulmonary sepsis [29].

### Definitions of ARDS, and non-pulmonary organ failures

ARDS and ARDS severity (mild, moderate, severe) were assessed daily using the Berlin definition [33], based on consensus review of all chest radiographs, blood gases, and clinical data by two physician investigators. If no blood gases were available, then the SpO_2_/FiO_2_ ratio was used to assess for the presence and severity of ARDS [34]. Patients who met criteria for ARDS for at least two consecutive study days were considered to have ARDS; patients who met ARDS criteria on only 1 day or on two non-consecutive days were considered to have an indeterminate ARDS status and were excluded from the study. Patients who did not meet criteria for ARDS on any of the 4 study days were included in the non-ARDS group. Patients with evidence of a primary cardiogenic cause for pulmonary edema were excluded from this study [35]. Definitions of circulatory, coagulation, hepatic and renal failures were based on the Brussels criteria [36]. Mortality analyses were conducted using in-hospital mortality as an endpoint.

### Biomarker selection and assays

Because endothelial glycocalyx dysfunction has been associated with acute lung injury in animal models of sepsis [24] and syndecan-1 is a well-documented marker of endothelial injury [37], we selected syndecan-1 as our primary marker of damage to the endothelial glycocalyx.

Plasma levels of syndecan-1 and myeloperoxidase were measured in duplicate in thawed citrated plasma samples collected on the morning of ICU day 2 at the time of enrollment into the VALID study using commercially available ELISA kits (Syndecan-1 Item No. ab46506, Abcam, Cambridge, MA, USA) or electrochemiluminescent immunoassays (Myeloperoxidase, Meso Scale Discovery, Rockville, MD, USA).

### Statistical analysis

Categorical variables are reported as frequencies (percentages). For clinical characteristics of the cohort, continuous variables were normally distributed and are reported as means with standard deviation, and groups were compared by *T* test or Chi-square analysis. For the analysis of biomarker levels which were not normally distributed, continuous variables are reported as medians (lower and upper quartiles) and groups were compared by Mann–Whitney *U* test. Correlation between variables was measured using Spearman’s *ρ*. For biomarker levels, the association between biomarker quartile and the outcome of interest was assessed by linear-by-linear association. To determine whether syndecan-1 levels were independently associated with in-hospital mortality, multivariable logistic regression was done and included age and APACHE II score, which are independent predictors of mortality, as covariates [31, 38–41]. Statistical analyses were conducted in SPSS Statistics version 24 (IBM, Armonk, NY).

## Results

### Patient characteristics

We studied 262 critically ill subjects with severe sepsis. Patient demographic and clinical characteristics are summarized in Table 1. Among this group, 127 (48%) had non-pulmonary sepsis, and 135 (52%) had pulmonary sepsis. Compared to patients with pulmonary sepsis, those with non-pulmonary sepsis were less likely to develop ARDS (44 vs. 59%, *p* = 0.03), more frequently required vasopressors (64 vs. 51%, *p* = 0.05) but had similar hospital mortality (38 vs. 34%, *p* = 0.61).

**Table 1:** Clinical characteristics of 262 critically ill medical ICU patients with severe sepsis who were included in the study

	All patients *N* = 262	Non-pulmonary sepsis *N* = 127	Pulmonary sepsis *N* = 135	*p* value
Age (years)	56 ± 16	56 ± 15	56 ± 17	0.98
Male	137 (52%)	63 (50%)	74 (55%)	0.46
Caucasian	224 (86%)	107 (84%)	117 (87%)	0.60
Ever smoker	149 (57%)	68 (54%)	82 (61%)	0.26
Alcohol abuse	55 (21%)	26 (21%)	30 (22%)	0.77
APACHE II	34 ± 6	34 ± 6	33 ± 6	0.06
Primary site of infection				
Lung			134 (100%)	
Abdomen		32 (25%)		
Urinary tract		23 (18%)		
Skin/soft tissue/bone		17 (13%)		
CNS/sinus		17 (13%)		
Endocarditis/catheter		25 (20%)		
Other or unclear		13 (10%)		
Required vasopressors		81 (64%)	69 (51%)	0.05
Developed ARDS	135 (52%)	56 (44%)	79 (59%)	0.03
Ventilator-free days 150 (57%)	14 ± 11	14 ± 11	14 ± 11	0.98
Died in hospital	94 (36%)	48 (38%)	46 (34%)	0.61

Data as mean ± SD or *n* (%) as indicated. *p* values compare characteristics of groups with pulmonary versus non-pulmonary sepsis by *T* test or Chi-square analysis as appropriate

### Syndecan-1 levels and risk of ARDS

**Fig. 1:**
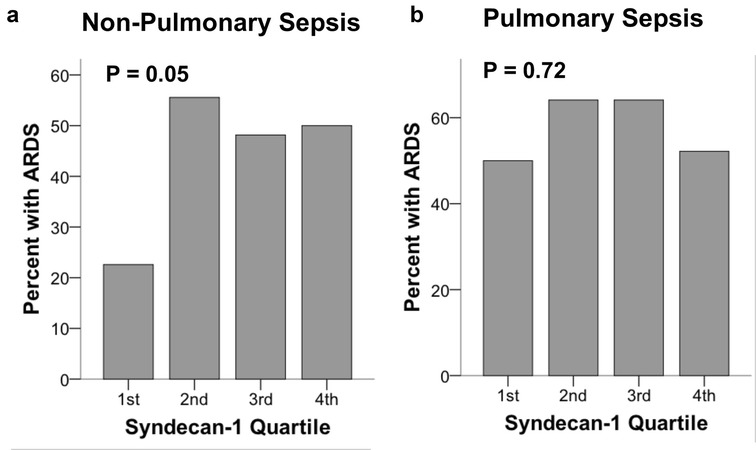
Higher plasma syndecan-1 levels by quartile were associated with development of ARDS in patients with non-pulmonary sepsis (**a**) but were not associated with ARDS in patients with pulmonary sepsis (**b**). ARDS was compared across quartiles by linear-by-linear association test

In the entire cohort, syndecan-1 levels were not significantly associated with diagnosis of ARDS (*p* = 0.17 for linear-by-linear association across quartiles of plasma syndecan-1 levels). However, when only patients with non-pulmonary sepsis were considered, higher syndecan-1 levels were significantly associated with ARDS (*p* = 0.05, Fig. 1a). ARDS was diagnosed in 50% of patients in the highest quartile of syndecan-1 levels compared to 22% in the lowest quartile (*p* = 0.028). There was no significant association of syndecan-1 levels with ARDS in the group of patients with pulmonary sepsis (*p* = 0.72, Fig. 1b).

**Fig. 2:**
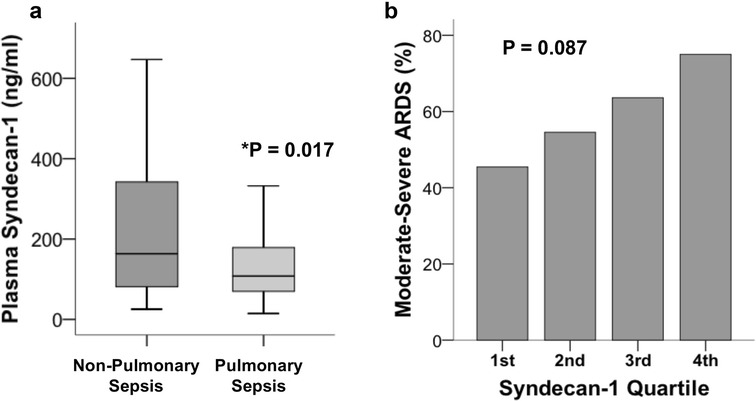
Among patients with ARDS (*N* = 135), plasma syndecan-1 levels were significantly higher in patients with non-pulmonary sepsis compared to those with pulmonary sepsis (**a**, *p* = 0.017 by Mann–Whitney *U* test). There was a trend for higher syndecan-1 levels to be associated with severity of ARDS in the subgroup (*n* = 56) of ARDS patients with non-pulmonary sepsis (**b**, *p* = 0.087 by linear-by-linear association test)

Among patients with ARDS, plasma syndecan-1 levels were significantly higher in patients with indirect ARDS due to non-pulmonary sepsis (*n* = 56) than in patients with direct ARDS due to pulmonary sepsis (*n* = 79) (*p* = 0.017, Fig. 2a). There was a trend toward association of higher syndecan-1 levels with greater ARDS severity (percent of patients with moderate or severe ARDS), but only in the subgroup of ARDS patients with ARDS due to non-pulmonary sepsis (Fig. 2b).

### Syndecan-1 levels and non-pulmonary organ failures

**Fig. 3:**
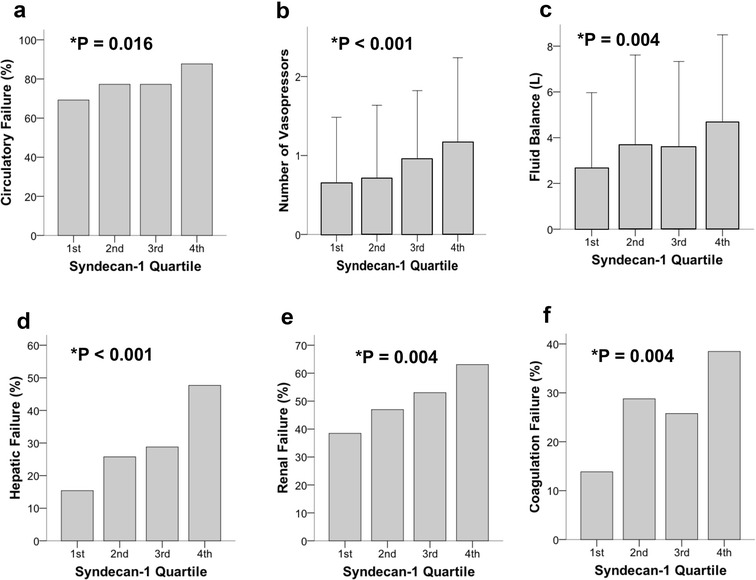
Higher plasma syndecan-1 levels by quartile were associated with non-pulmonary organ dysfunction at enrollment including **a** circulatory failure, **b** number of vasopressors, **c** fluid balance over the first 24 h, **d** hepatic failure, **e** renal failure, **f** coagulation failure. Organ failures were defined by Brussels organ failure scores at enrollment. *p* values by linear-by-linear association test except for number of vasopressors and fluid balance which were analyzed by linear regression

Higher plasma syndecan-1 levels by quartile were significantly associated with the presence of circulatory failure as defined by Brussels Organ Failure Scoring (Fig. 3a, *p* = 0.016), number of vasopressors required (Fig. 3b, *p* < 0.001), or fluid balance over the first 24 h (Fig. 3c, *p* = 0.004). When patients were grouped by need for any vasopressor at enrollment, syndecan-1 levels were significantly higher in those requiring vasopressors (median 157, IQR 84–306 vs. median 84, IQR 52–180, *p* < 0.001). Higher plasma syndecan-1 levels by quartile were also significantly associated with hepatic failure (Fig. 3d, *p* < 0.001), renal failure (Fig. 3e, *p* = 0.004), and coagulation failure (Fig. 3f, *p* = 0.004) at study enrollment as defined by Brussels organ failure scoring. These analyses were conducted in the entire patient cohort (*n* = 262). By contrast, plasma lactate levels were not significantly associated with non-pulmonary organ dysfunction (data not shown), although clinically measured levels were only available in a subset of patients (*n* = 74). Lactate levels and syndecan-1 levels were also not significantly correlated (Spearman’s *ρ* = 0.181, *p* = 0.12).

### Syndecan-1 levels and clinical outcomes

**Fig. 4:**
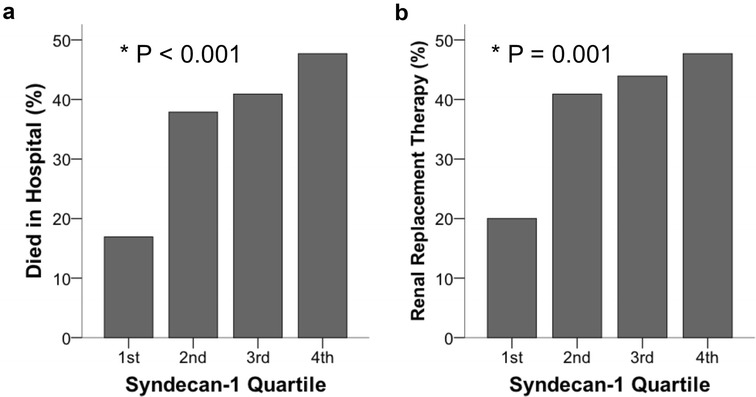
Clinical outcomes including **a** hospital mortality and **b** need for renal replacement therapy were strongly associated with increasing plasma syndecan-1 levels in 262 patients with sepsis. *p* values by linear-by-linear association test

In the entire cohort, patients with higher plasma syndecan-1 levels were significantly more likely to die during hospitalization (Fig. 4a, *p* < 0.001) and to require renal replacement therapy during hospitalization (Fig. 4b, *p* = 0.001). In-hospital death was much more frequent in patients in the highest quartile of syndecan-1 (48%) compared to patients in the lowest quartile (17%, *p* < 0.001). Higher syndecan-1 levels were also independently predictive of in-hospital mortality in a multivariable logistic regression model adjusted for age and APACHE II score (Table 2; odds ratio 1.85 per log increase in syndecan-1, 95% CI 1.056–3.241).

**Table 2:** Multivariable logistic regression model for hospital mortality in 262 sepsis patients

Variable	OR (95% CI)	*p* value
Syndecan-1 (per log increase)	1.850 (1.056–3.241)	0.031
Age (per year increase)	1.009 (0.992–1.026)	0.309
APACHE II (per one point increase)	1.035 (0.991–1.082)	0.125

### Syndecan-1 levels and neutrophil activation

To determine whether syndecan-1 shedding was associated with evidence of systemic neutrophil activation, we measured plasma MPO levels in all patients who had sufficient plasma available after syndecan-1 measurements (*n* = 247). Plasma MPO levels were only weakly associated with syndecan-1 levels (Spearman’s *ρ* = 0.21, *p* < 0.001). In patients with non-pulmonary sepsis, the association was somewhat stronger (Spearman’s *ρ* = 0.294, *p* < 0.001). Syndecan-1 levels were not associated with total white blood cell count (*ρ* = 0.064, *p* = 0.3).

## Discussion

Degradation of the endothelial glycocalyx has been increasingly recognized as an important contributor to the pathophysiology of sepsis [13]. Several studies have demonstrated elevated syndecan-1 levels as a marker of glycocalyx degradation in patients with sepsis. In a study of 150 patients, Steppan et al. [9] compared patients with sepsis and patients after major abdominal surgery to controls and found highest levels of syndecan-1 and inflammatory markers in sepsis patients. Two other small observational studies of eighteen [42] and twenty [11] patients with septic shock demonstrated elevated syndecan-1 levels compared to controls. However, to our knowledge, this is the first large-scale study of syndecan-1 as a biomarker of risk of ARDS and other organ dysfunction in patients with sepsis. Similarly, it is the first study to assess the independent association of this biomarker with sepsis mortality. Our findings suggest that in patients with sepsis, the severity of glycocalyx degradation, as measured by syndecan-1, is strongly associated with organ dysfunction and mortality, but contrary to our primary hypothesis, is only associated with development of ARDS in patients with non-pulmonary sepsis.

The finding of higher syndecan-1 levels in patients with ARDS due to non-pulmonary sepsis compared to ARDS due to pulmonary sepsis suggests that degradation of the endothelial glycocalyx may be more prominent in the pathophysiology of non-pulmonary sepsis. Concordant with this finding, elevated syndecan-1 levels were only associated with development of ARDS in patients with non-pulmonary sepsis. These observations are concordant with our prior observation that other biomarkers of endothelial injury such as angiopoietin-2 and von Willebrand factor antigen are more elevated in non-pulmonary sepsis patients with indirect ARDS compared to pulmonary sepsis patients with direct ARDS [24]. Similarly, the predominance of heparan sulfate fragments in indirect respiratory failure reported by Schmidt et al. [29] likely indicates a greater degree of glycocalyx degradation in that patient group. Taken together, these findings provide strong evidence that injury to the endothelial barrier is more severe in indirect mechanisms of acute lung injury than in direct mechanisms. These findings may explain, in part, why a recent study of a large group of patients found that in contrast to direct ARDS, where age and the severity of ARDS were independent predictors of mortality, in indirect ARDS, only the number of non-pulmonary organ failures was independently associated with mortality [43].

The diagnosis of ARDS is still based on clinical criteria and does not rely on underlying pathophysiology [33]. As a result, current definitions identify a highly heterogeneous group of patients that may have different underlying mechanisms of acute lung injury and different responses to therapy. Using a latent class analysis approach, Calfee and colleagues reported that distinct subphenotypes of ARDS can be identified that respond differently to experimental therapies [44, 45]. The current findings provide further evidence of the heterogeneity of ARDS in humans as defined by the extent of endothelial glycocalyx degradation. If validated, syndecan-1 might be useful as a biomarker to help further distinguish molecular phenotypes of ARDS.

Alternatively, plasma syndecan-1 levels might be used to identify subgroups of sepsis patients for therapy targeted at protection or restoration of the endothelial glycocalyx. In patients with severe trauma and hemorrhagic shock, glycocalyx shedding causes coagulopathy and perturbations in fibrinolysis [8]. Furthermore, recent evidence suggests that transfusion of plasma reduces syndecan-1 shedding and reconstitutes the glycocalyx in hemorrhagic shock [14]. Only 22 patients in the current study received transfusion of fresh frozen plasma prior to blood sampling, and we did not find any relationship between receipt of plasma and syndecan-1 levels (data not shown); however, our power for this analysis was low. Another therapy that may affect the glycocalyx is sevoflurane, which has been shown to reduce glycocalyx shedding and leukocyte adhesion in animal models of ischemia–reperfusion injury [46–48].

This study has several strengths including the large sample size and detailed prospective phenotyping for ARDS and other organ dysfunction as part of the parent VALID cohort study. There are also some limitations. First, the observed association between syndecan-1 shedding and organ dysfunction and clinical outcomes does not prove causation, nor does it elucidate the underlying mechanisms by which glycocalyx shedding contributes to organ dysfunction. Although we hypothesized that neutrophil activation contributes to enzymatic cleavage of syndecan-1 from the endothelial cell surface, we did not find a strong correlation between MPO, a circulating marker of neutrophil activation and syndecan-1 levels. Second, we intentionally selected patients who were more severely ill and more likely to develop ARDS by requiring an APACHE II score of 25 or greater and mechanical ventilation. These inclusion criteria were selected to maximize the likelihood of studying patients with a significant degree of glycocalyx degradation. However, the findings may not be generalizable to a less severely ill patient population, nor are the findings necessarily applicable to patients at risk of ARDS from non-septic causes or to patients with surgical critical illness. Finally, it is not possible to know from the current study what fraction of elevated syndecan-1 levels is due to increased shedding of the glycocalyx, versus impaired clearance of syndecan-1. If syndecan-1 were cleared primarily in the liver or kidney, then the association between elevated levels and kidney and liver dysfunction might simply reflect impaired clearance. However, very little is known about the clearance of syndecan-1 from the circulation. In one study of patients with chronic kidney disease, plasma syndecan-1 clearance was not a function of creatinine clearance [49].

In summary, the current findings highlight the potential importance of disruption of the endothelial surface layer in the pathogenesis of organ dysfunction in sepsis. Further studies are warranted to validate the current findings in other patient populations and to determine the precise mechanisms of organ injury that occurs in association with glycocalyx degradation. In addition, this study adds to the growing body of evidence that sepsis and ARDS are not homogenous disease states. Further characterization of molecular phenotypes of organ dysfunction and acute lung injury in sepsis may help to better target therapies in the future, including therapies targeted at protection and restoration of the endothelial glycocalyx.

## Conclusions

In conclusion, our study evaluated the severity of endothelial glycocalyx degradation and its impact on ARDS, non-pulmonary organ dysfunction, and mortality in a cohort of critically ill patients with sepsis. Contrary to our initial hypothesis, elevated syndecan-1 levels were associated with ARDS only in a subgroup of patients with non-pulmonary sepsis, suggesting that degradation of the glycocalyx is more severe in patients with non-pulmonary sepsis, and adding to the growing body of evidence that the mechanisms underlying direct and indirect causes of ARDS are distinct. Regardless of etiology of sepsis, elevated syndecan-1 levels were associated with non-pulmonary organ dysfunction and in-hospital mortality. Together, these findings suggest that measurement of syndecan-1 levels in patients with sepsis may be useful for identifying patients at high risk of organ dysfunction and mortality and those who may benefit from therapies targeted at protecting or restoring the glycocalyx.

Ethics approval and consent to participate: The VALID study was approved by the Vanderbilt Institutional Review Board (#051065). Informed consent was obtained from patients or their surrogates prior to enrollment. Due to the minimal risk of this observational study, a waiver of informed consent was granted by the Institutional Review Boards for patients who were unable to participate in the informed consent process and for whom no surrogate decision maker was available.

## Abbreviations

AECC: American-European consensus conference
APACHE II: acute physiology and chronic health evaluation II
ARDS: acute respiratory distress syndrome
ELISA: enzyme-linked immunosorbent assay
ESL: endothelial surface layer
MPO: myeloperoxidase
VALID: validating biomarkers of acute lung injury for diagnosis
